# Systematic integration of machine learning algorithms to develop immune escape-related signatures to improve clinical outcomes in lung adenocarcinoma patients

**DOI:** 10.3389/fimmu.2023.1131768

**Published:** 2023-03-02

**Authors:** Ting Wang, Lin Huang, Jie Zhou, Lu Li

**Affiliations:** Lung Cancer Center, West China Hospital, Sichuan University, Chengdu, China

**Keywords:** immune checkpoint inhibitors, immunothearpy, immune escape, machine learning (ML), lung adenocarcacinoma

## Abstract

**Background:**

Immune escape has recently emerged as one of the barriers to the efficacy of immunotherapy in lung adenocarcinoma (LUAD). However, the clinical significance and function of immune escape markers in LUAD have largely not been clarified.

**Methods:**

In this study, we constructed a stable and accurate immune escape score (IERS) by systematically integrating 10 machine learning algorithms. We further investigated the clinical significance, functional status, TME interactions, and genomic alterations of different IERS subtypes to explore potential mechanisms. In addition, we validated the most important variable in the model through cellular experiments.

**Results:**

The IERS is an independent risk factor for overall survival, superior to traditional clinical variables and published molecular signatures. IERS-based risk stratification can be well applied to LUAD patients. In addition, high IERS is associated with stronger tumor proliferation and immunosuppression. Low IERS exhibited abundant lymphocyte infiltration and active immune activity. Finally, high IERS is more sensitive to first-line chemotherapy for LUAD, while low IERS is more sensitive to immunotherapy.

**Conclusion:**

In conclusion, IERS may serve as a promising clinical tool to improve risk stratification and clinical management of individual LUAD patients and may enhance the understanding of immune escape.

## Introduction

Lung cancer is the most commonly diagnosed malignancy in the world and is one of the leading causes of cancer-related deaths (1). Lung cancer is highly heterogeneous, of which lung adenocarcinoma (LUAD) is the most common subtype, accounting for approximately 47% of all lung cancer cases (2). LUAD is highly aggressive and malignant, leading to high prevalence and mortality rates, with high mortality rates largely attributable to disease progression and inappropriate treatment (3). The prognosis of LUAD varies widely among patients depending on the stages at the time of diagnosis (4). Despite innovations in surgery, chemotherapy, and targeted therapies for lung cancer over the past decades, the prognosis of patients with advanced and some early-stage LUAD remains unsatisfactory (4). Recently, immune blockade therapies targeting PD-1, PD-L1, and other immune checkpoints are emerging as a new hope for the treatment of cancer patients (5). Although immune blockade therapies have improved the overall prognosis of patients with LUAD as well as recently reported, only about 15% of patients benefit from them (6). The identification of patients with early-stage LUAD and the limited efficacy of immunotherapy has become the major barrier preventing further improvement in the prognosis of LUAD.

The immune system and the tumor microenvironment (TME) are critical regulators of the tumor system and have both supportive and inhibitory properties in cancer development, progression, and invasion (7). Revolutionary therapies targeting the immune system in the TME are the recent focus of several promising therapeutic approaches for cancer patients (8). The central step in cancer immunotherapy is the active recognition of the malignant transformation of cells by the immune system and the mobilization of effector cells to clear the malignant cells (9). This step relies not only on the immunogenicity of the tumor cells themselves but also on non-mutated and mutated antigens on the surfaces of the tumor cells. However, recent studies have shown that immune escape occurs during cancer progression, exempting tumor cells from immune clearance, leading to poor immunotherapy responses and poor patient prognosis. On the one hand, tumor cells avoid recognizing cancer-specific antigens by the immune system through cloning and evolution of their genome profiles (10); on the other hand, tumor cells also summon immunosuppressive cells to suppress immunogenicity and escape clearance (11). Thus, despite the demonstrated potential of using the immune system to clear tumors, the clinical reality of TME is often an environment of immune escape. Recent advances in large-scale transcriptome sequencing technology and molecular biology have helped us explore the dynamics of tumors and TME in greater depth. We can rely on this solid bridge to better understand the mechanisms of immune escape to improve immunotherapy and tumor prognosis.

In this study, we attempted to apply immune escape-related markers based on multiple machine learning algorithms to develop and validate risk stratification characteristics–immune escape-related scores (IERS) for a total of 1107 LUAD patients from 4 datasets in the TCGA and GEO databases. We then evaluated the association of IERS with biological function, TME, and genomic mutations. Finally, we evaluated the benefit of IERS in predicting first-line chemotherapy and immunotherapy in patients with LUAD. This work may help to enhance the understanding of immune escape and optimize clinical decision-making and prognosis in patients with LUAD.

## Methods

### Collection and processing of public datasets

The TCGA-LUAD dataset from 492 LUAD patients containing detailed clinical information and follow-up data was accessed using UCSC Xena (https://xena.ucsc.edu/). We collected RNA-seq data, maf data from the Mutect2 platform, copy number variation (CNV) data after gistic2.0 processing, and Illumina 450 methylation data of TCGA-LUAD. Three GEO datasets were accessed and collected from the GEO database: GSE30219, GSE42127, and GSE72094. after excluding patients with incomplete clinical information and other pathological types, a final GEO meta-cohort of 615 LUAD patients was obtained. In addition, two datasets (Imvigor210 and Nature-SKCM) were collected: containing 298 patients with bladder cancer treated with anti-PD-L1 and 121 patients with melanoma treated with anti-PD-1, respectively (12, 13). These two immunotherapy-related datasets were used to assess the efficacy of IERS in predicting response to immunotherapy.

The raw RNA-seq datasets from TCGA were converted to transcript matrices in kilobases per million (TPM) and further normalized by log2 transformed. The GEO meta-cohort was obtained from three different platforms: GPL570, GPL6884, and GPL15048. The transcripts from the three datasets were processed and merged using the COMBAT function from the “sva” package to remove batch effects between chips (14). Ultimately, the TCGA-LUAD cohort was used to filter features and construct models and the GEO meta-cohort was used to test and validate the final model. We extracted the set of genes regulating immune escape from the previous study by Lawson et al. (15).

### Pipeline for generating risk signatures based on machine learning

To generate stable and highly accurate immune escape correlation scores (IERS), we used the following steps: (a) univariate Cox regression analysis to identify immune escape genes with independent prognostic efficacy; (b) we integrated 10 machine learning algorithms including stepwise Cox, CoxBoost, Cox partial least squares regression (plsRcox), generalized augmented regression model (GBM), Stochastic Survival Forest (RSF), Elastic Network (Enet), Lasso, Ridge, Supervised Principal Component (SuperPC), and Survival Support Vector Machine (survival-SVM). One algorithm was used to filter the variables, and another was used to construct the model, and the final fit of 101 algorithm combinations was evaluated and prevented from overfitting by leave-one-out cross-validation (16). (c) Validation of the predictive effect of the final generated models in the Meta-GEO cohort. The best model was determined by calculating the C-index of all models in different cohorts, with a higher C-index indicating a more stable model (17).

### Functional enrichment and immune infiltration analysis

Functional enrichment of genes was achieved through the Metascape website (https://metascape.org/gp), and GSEA software (version 4.1.0) was used to assess the differences in KEGG pathways between subgroups. The ssGSEA algorithm in package “gsva” is used to assess the relative activity of the pathway of interest, and a detailed list of pathway genes was provided in **Table S1**. We used “cibersort” to assess the relative infiltration abundance of 22 immune cell types by matching transcripts (18). The tumor purity and immune score of individual samples were assessed by the Estimate algorithm (19). Finally, we collected Homologous recombination defects (HRD) scores, indel neoantigens, and SNV neoantigens from the TCGA-LUAD cohort from previous studies (20).

### Analysis of maf data

We used “maftools” to process and analyze the raw maf data (21). We first identified the driver mutation factors (mutation frequency >45) and examined differences in driver mutations between high and low IERS subgroups. Subsequently, we calculated the nonsynonymous mutational load and estimated its correlation with IERS. Finally, we extracted and summarized significant mutation signatures for different subgroups from the maf data through the “Sigminer” package and annotated each mutation signature by comparison with the COSMIC database (22). Furthermore, CNV data were visualized by ggplot2.

### Prediction of treatment response

We used the R package “pRRophetic” based on the 2016 version of the GDSC database to predict the sensitivity of individual patients to chemotherapy drugs, and calculated IC50 values by ridge regression, with lower IC50 values indicating higher sensitivity (23). In addition, differential genes for top150 between high and low IERS subgroups were submitted to the Cmap database (https://clue.io/) to predict possible small molecule compounds. For the immunotherapy response, we first calculated the Immunophenoscore (IPS) of the samples based on a previous algorithm, with higher IPS suggesting stronger immune activity and stronger immunotherapy response (24). The TIDE algorithm (http://tide.dfci.harvard.edu) was then used to predict the difference in response rates between the high and low IERS subgroups to immunotherapy, and the accuracy was assessed by recipient operating characteristic (ROC) curves (25–28). Finally, IERS was generated and assessed for accuracy in the two previously mentioned immunotherapy cohorts.

### Statistical analysis

All statistical analyses, data processing, and graphing were performed in R 4.1.3 software. Continuous variables between the two groups were tested by t-test or Wilcoxon test, depending on the case, and categorical variables were tested by chi-square test. Correlations of continuous variables were assessed by the spearman correlation coefficient. Cox regression, Kaplan-Meier analysis, and nomogram generation were performed using the “rms” package and the “survival” package. and time-dependent ROC curves were constructed using the “pROC” and “timeROC” packages, respectively. If not mentioned otherwise, statistical tests are two-sided and P<0.05 is considered statistically significant.

### Cellular experiments *in vitro*

The normal human bronchial epithelial cell line 16HBE and the human lung adenocarcinoma cell line A549 was bought from Shanghai EK Bioscience Co. All cells were grown in DMEM media with 10% FBS in a 37°C cell incubator with 5% CO2. We then used qRT-PCR to assay normal and tumor cell lines to assess FADD level. The real-time PCR experiment (Vazyme, China) was performed by ChamQ Universal SYBR qPCR Master Mix. We used GAPDH as a control, then the amplified PCR products were measured and standardized. The primer sequences were: FADD, forword 5’GAGAAG GAGAACGCAACA-3’; Reverse 5’-GACGCTTCGGAGG TAGAT-3’. The LipofectamineTM 2000 Transfection Reagent (Invitrogen, USA) was used for the transfection of siRNA in this work. Cell Counting Kit-8 kit (Bioss, China) was used to measure the proliferation rate of normal and tumor cell line. We selected three chambers in different groups at 0, 12, 24, 48, and 72 hours respectively. Then, 10 mL of the Cell Counting Kit-8 reagent was added, and the wells were incubated at 37°C for two hours. identification of 450 nm absorbance values. Transwell kit (Merck Millipore, USA) was used to detect the invasive ability of different cell lines. We conducted experiments according to the manufacturer’s instructions and then performed cell counts by ImageJ software.

## Results

### Characterization of immune escape in TCGA-LUAD

**Figure 1:**
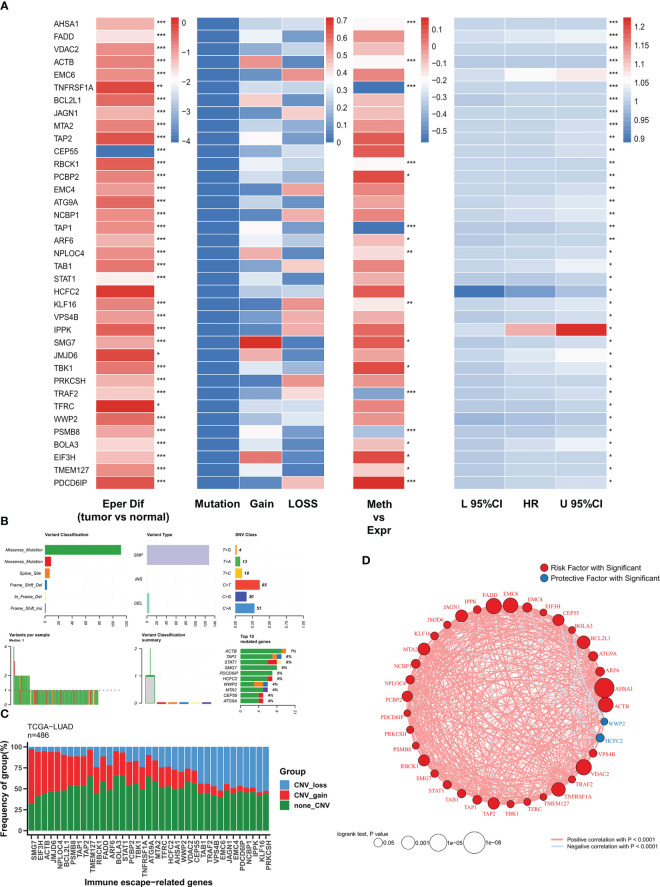
The genomic profiles of immune escape genes in TCGA-LUAD **(A)** Multi-omics transcriptome profiles of immune escape genes in TCGA-LUAD. From left to right: analysis of variance, genomic alterations, methylation correlation, univariate Cox regression. **(B)** Summary of single nucleotide mutations of immune escape genes in TCGA-LUAD. **(C)** Summary of copy number variations of immune escape genes in TCGA-LUAD. **(D)** Correlation network of immune escape genes. :*P<0.05, **P<0.01, ***P<0.001.

We first analyzed the expression, genomic alterations, methylation, and prognostic efficacy of immune escape genes in the TCGA-LUAD cohort. **Figure 1A** summarizes the transcriptomic features of 37 immune escape genes with independent prognosis. Except for CEP55, immune escape genes were highly expressed in tumor samples. Immune escape genes showed few single nucleotide variants (SNV) in TCGA-LUAD, with a high frequency of CNV events, which may play a major regulatory role. TNFRSF1A, TAP1, TRAF2, and PSMB8 were significantly negatively correlated with methylation levels, suggesting that methylation is their main modality of regulation. Except for HCFC2 and WWP2, immune escape genes were risk factors. The major mutation mode of these genes was nonsense mutation, and ACTB was the most frequently mutated gene (7%) (**Figure 1B**). CNV was the major genomic alteration mode of 37 immune escape genes, SMG7, EIF43H and ACTB were the most frequently amplified genes, and KLF16 and PRKCSH were the most frequent deletion genes (**Figure 1C**). **Figure 1D** demonstrates their correlation network with a high positive correlation between risk factors, while HCFC2 and WWP are negatively correlated with other genes.

### Integrative construction of robust risk stratification signatures

**Figure 2:**
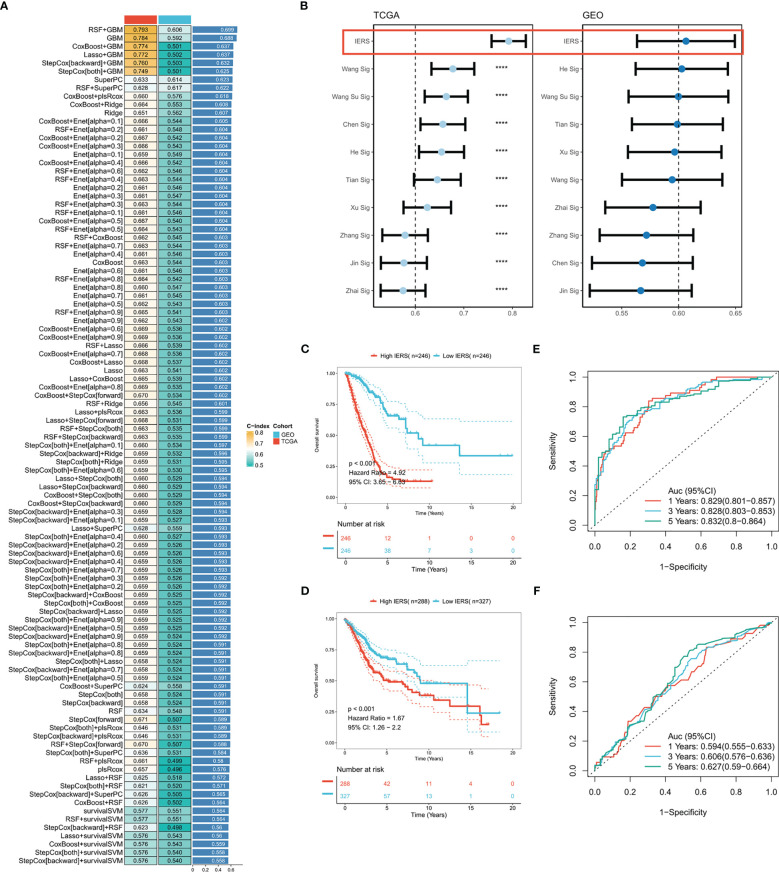
Systematic integration of machine learning algorithms to construct IERS **(A)** C index was calculated after cross-validation for a total of 101 algorithm combinations in both TCGA and GEO cohorts. **(B)** Comparing the accuracy of IERS with 10 published molecular signatures. **(C)** KM survival curves for the high-IERS and low-IERS groups in the TCGA cohort. **(D)** KM survival curves for the high-IERS and low-IERS groups in the meta-GEO cohort. **(E)** 1-, 3-, and 5-year ROC curves for IERS in the TCGA cohort. **(F)** 1-, 3-, and 5-year ROC curves for IERS in the meta-GEO cohort. ****P<0.0001.

Based on 37 prognostic immune escape genes, we performed a composite machine learning pipeline to generate robust immune escape-related scores (IERS). We fitted 101 machine learning combinations in the TCGA-LUAD cohort and validated them in the meta-geo cohort. Based on the average C index we found that the algorithm combination of RSF+GBM generates the best model with a leading edge (**Figure 2A**). Interestingly, the GBM algorithm alone also gives good results (**Figure 2A**). In addition, we retrieved 10 public mRNA signatures of LUAD that are associated with various biological processes (autophagy, ferroptosis, metabolism, etc.). We calculated the C index of the public signatures and found that our model has a leading advantage over the previous model (**Figure 2B**). Patients at high and low risk were divided based on the median of IERS, and the results showed significantly better survival in patients with low IERS in the TCGA and GEO cohorts (**Figures 2C, D**). the ROC curves showed superior efficacy of IERS in the TCGA cohort (1 year: 0.829, 3 years: 0.828, 5 years: 0.832) (**Figure 2E**). In contrast, IERS underperformed in the GEO cohort (1 year: 0.594, 3 years: 0.606, 5 years: 0.627) (**Figure 2F**).

### Generation of individual IERS risk stratification

**Figure 3:**
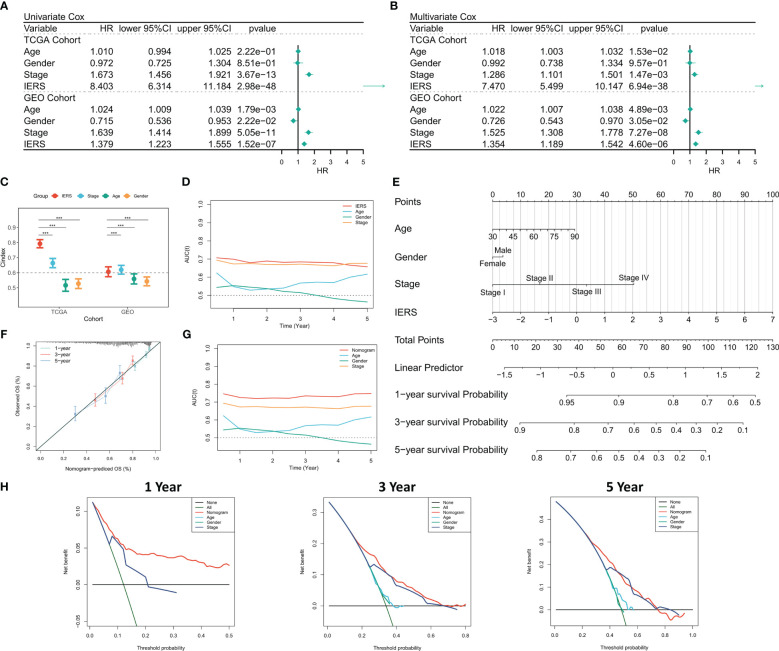
Evaluation of the IERS model **(A)** Univariate Cox regression analysis of OS in TCGA and meta-GEO cohorts; **(B)** Multivariate Cox regression analysis of OS in TCGA and meta-GEO cohorts. **(C)** Comparison of the performance of IERS and other clinical indicators. **(D)** timeROC curves for the IERS and clinical characteristics. **(E)** Construction of a nomogram based on the IERS. **(F)** Calibration curves for the nomogram. **(G)** timeROC curves for the nomogram and other clinical characteristics. **(H)** 1-, 3-, and 5-year DCA curves for the nomogram and other clinical characteristics. ***P<0.001.

Univariate Cox regression showed that IERS was an independent risk factor (**Figure 3A**). After correction for other clinical variables, IERS remained a significant risk factor (**Figure 3B**). We compared the efficacy of IERS with other clinical variables, with IERS showing a leading edge in the TCGA cohort and IERS slightly worse than Stage but better than other variables in the GEO cohort (**Figure 3C**). timeROC curves showed that IERS had similar performance to Stage in the total cohort of 1107 patients (**Figure 3D**). To better risk stratify and apply IERS to individual patients, we survived Nomogram (**Figure 3E**). Calibration curves showed good predictive efficacy of nomogram at 1, 3 and 5 years (**Figure 3F**). timeROC curves showed better predictive efficacy of the nomogram model than other clinical variables (**Figure 3G**). Finally, we evaluated the clinical benefit of the nomogram by DCA curves, and the results showed that the nomogram model had the best benefit at 1, 3, and 5 years (**Figure 3H**).

### Evaluation of IERS at single-cell resolution

**Figure 4:**
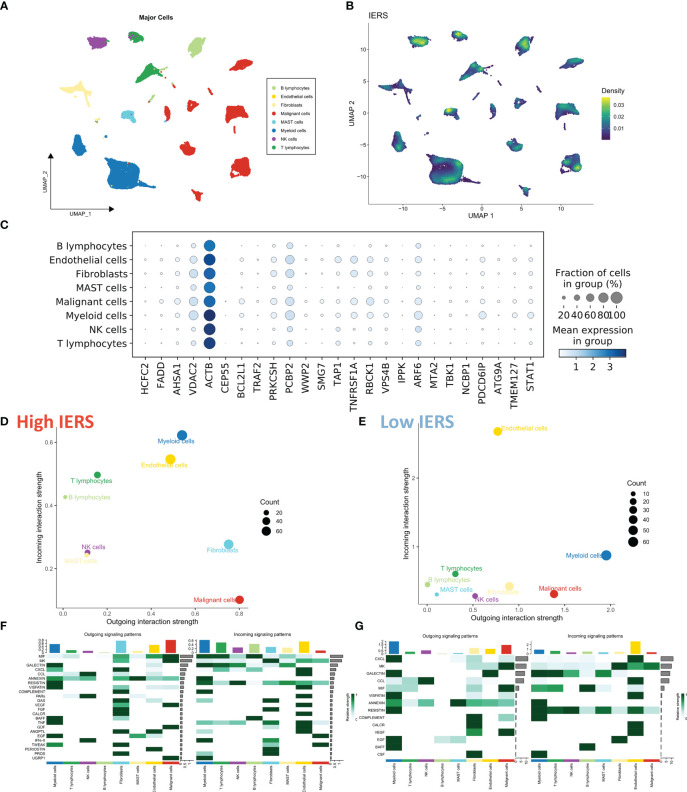
Evaluation of IERS at single-cell resolution **(A)** Eight identified cell clusters are shown based on Umap descending. **(B)** M Distribution of IERS in different cell clusters. **(C)** Expression of IERS model genes in different cell clusters. **(D)** Overall cellular communication intensity in high IERS cells. **(E)** Overall cellular communication intensity in low IERS cells. **(F)** Specific communication pathways between high IERS cells. **(G)** Specific communication pathways between low IERS cells.

We collected the single-cell dataset gse131907 and processed it with the “seurat” package to assess the distribution of IERS in TME (29). Based on the original annotation, a total of 8 major cell populations were identified (**Figure 4A**). We found that IERS was mainly distributed in the immune cell population as well as in some malignant cell populations (**Figure 4B**). In addition, the 25 immune escape genes that constitute the final model were also expressed mainly in the malignant and immune cell populations (**Figure 4C**). We then used the “cellchat” package to assess cellular communication between the high IERS and low IERS cell populations (30). We found that myeloid cells and malignant cells were more active in the high IERS group (**Figure 4D**). While in the low IERS group the stronger communication activity was myeloid cells and endothelial cells (**Figure 4E**). Malignant cells in the high IERS group mainly communicated through the VEGF and EGF pathways, and myeloid cells communicated through the TNF and MIF pathways (**Figure 4F**). In the low IERS group, endothelial cells received signals through CXCL and VEGF pathways, and myeloid cells mainly interacted through CXCL pathway, and the signal intensity was higher than that in the high IERS group (**Figure 4G**). Taken together, these results suggest that high IERS can distinguish between an escaped immune system and an immunosuppressed TME.

### Resolution of the biological function of IERS

**Figure 5:**
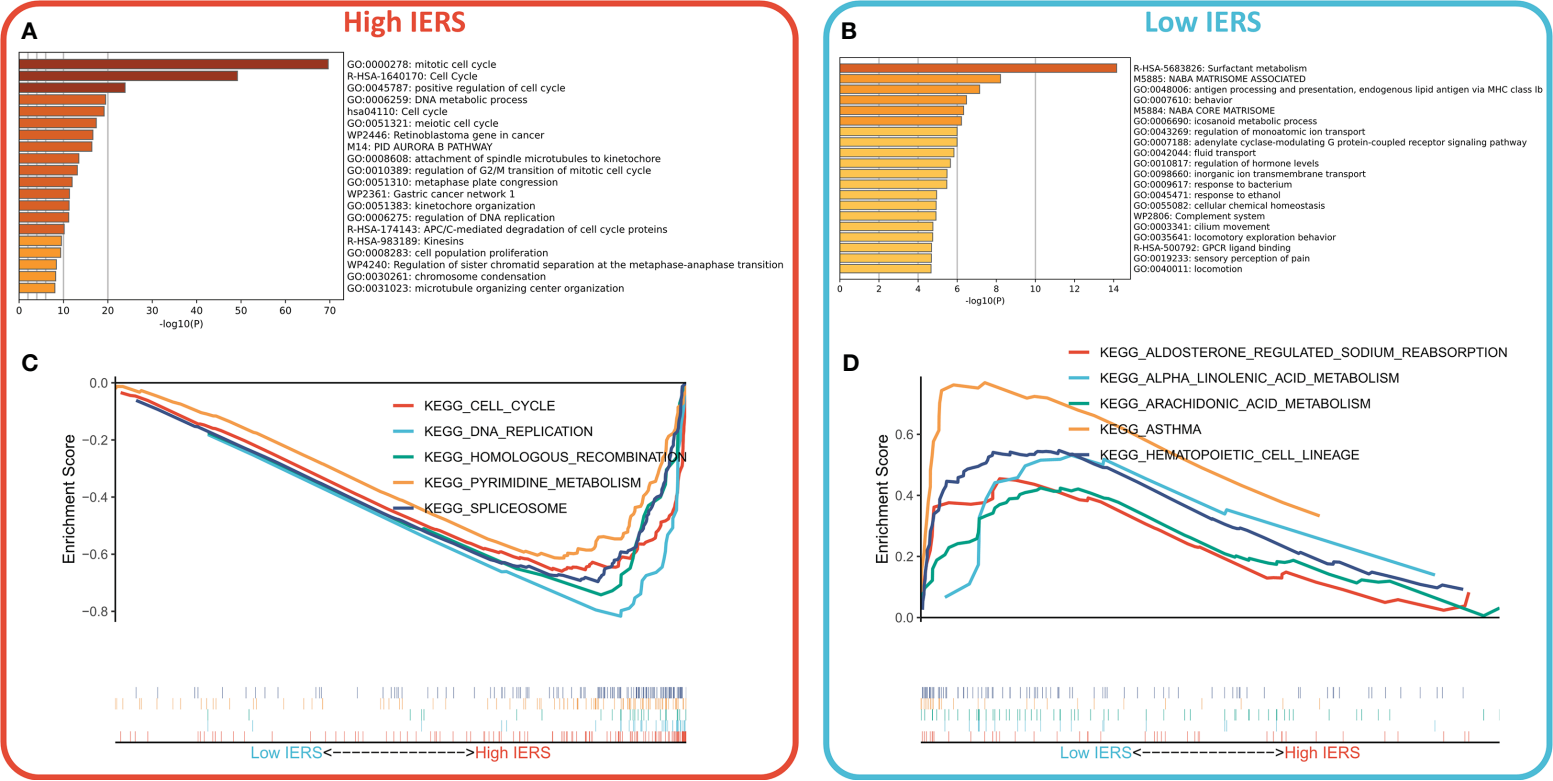
Functional analysis for IERS **(A)** Bar plot showed the biological pathways of upregulated gene enrichment in the high IERS group. **(B)** Bar plot showed the biological pathways of upregulated gene enrichment in the low IERS group. **(C)** GSEA analysis revealed the top five enriched KEGG pathways in the high IERS group. **(D)** GSEA analysis revealed the top five enriched KEGG pathways in the low IERS group.

We first identified genes that were highly expressed in the high and low IERS groups by a threshold of FC>2, adjust p value<0.05. Functional enrichment showed that the genes upregulated in the high IERS group were mainly associated with DNA metabolism, cell cycle, and cell proliferation (**Figure 5A**). And the genes upregulated in the low IERS group were mainly associated with antigen presentation and some regulatory pathways (**Figure 5B**). GSEA analysis showed that cell cycle, DNA replication, and homologous recombination pathways were upregulated in the high IERS group (**Figure 5C**). In contrast, asthma and hematopoietic cell line-related pathways were upregulated in the low IERS group (**Figure 5D**). In summary, we infer that tumor cell division and proliferation were active in the high IERS group, while immunoreactivity was stronger in the low IERS group.

### Classification of the tumor microenvironment by IERS

**Figure 6:**
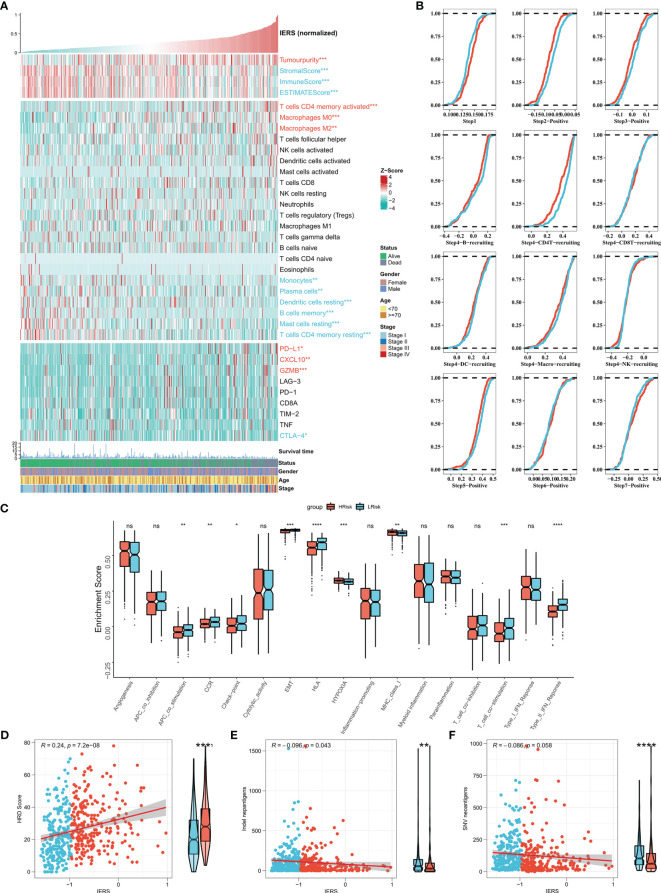
Dissecting the heterogeneity of TME among IERS subgroups **(A)** Heat map showed the IERS landscape, including the Estimate score, immune cell infiltration abundances, and immune checkpoint expression. **(B)** Cumulative distribution plots show differences in immune processes between different IERS subgroups. **(C)** The box plot showed the difference between different IERS subgroups for the interesting pathway calculated by the ssGSEA algorithm. The correlation between IERS and **(E)** HRD score, **(D)** Indel neoantigens, and **(F)** SNV neoantigens. ns P<0.05, *P<0.05, **P<0.01, ***P<0.001, ****P<0.0001.

We first summarized the association of IERS with tumor purity, immune cell abundance, and immune checkpoint expression to initially characterize TME in different IERS patients (**Figure 6A**). The results showed that high IERS was associated with high tumor purity, high activated CD4 memory cells, M0 and M1 macrophage abundance, high PD-L1, CXCL10, and GZMB expression. In contrast, low IERS was associated with higher immune scores, high monocyte, plasma cell, mast cell, and dendritic cell abundance, and high CTLA-4 expression. Further, we analyzed the differences in the distribution of the different tumor immunization steps in the high and low IERS groups. The results showed that the initial step was more abundant in the low IERS group, while step II and step 4-CD4 cell recruit were increased in the high IERS group (**Figure 6B**). It seems that the high IERS group is TME with CD4T cells as the main cells. Further, we evaluated the distribution of immune-related pathways among the different groups and the results showed that hypoxia and MHC class I pathways were more active in the high IERS group, while CCR, checkpoint, EMT, and some other immune-related pathways were more active in the low IERS group (**Figure 6C**). Finally, we found a positive correlation between IERS and HRD score (**Figure 6D**) and a significant negative correlation with indel neoantigens and SNV neoantigens (**Figures 6E, F**). In summary, patients with high IERS may face immunosuppressive TME. Whereas low IERS are immunologically active TME as well as having more neoantigens and may be more beneficial to immunotherapy.

### Dissecting genomic alterations between subgroups

**Figure 7:**
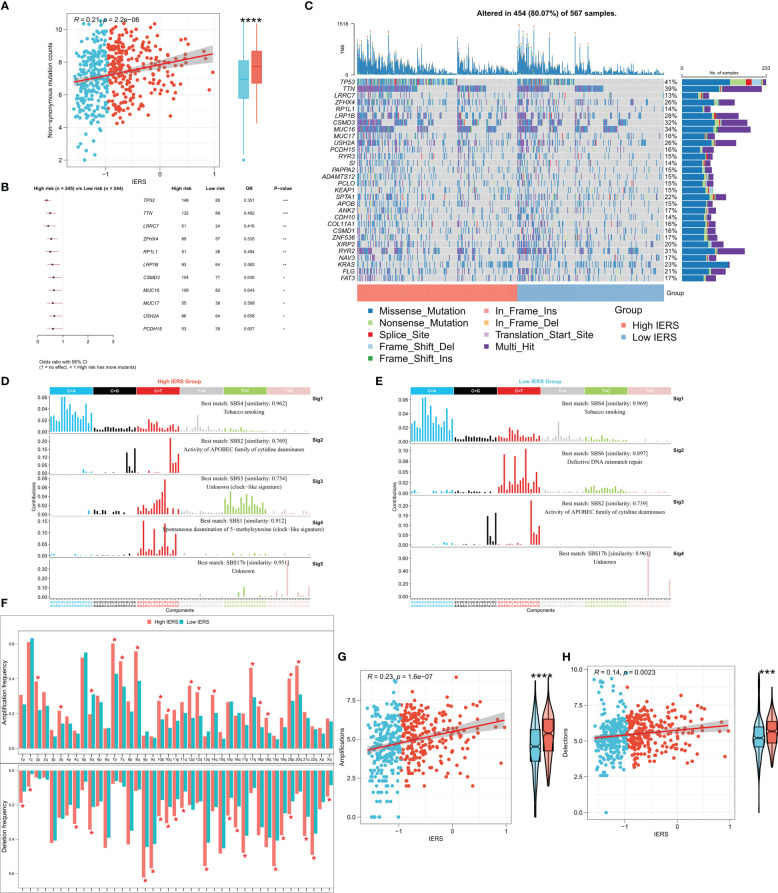
Genomic variation landscape of IERS **(A)** Box plots and scatter plots showed the correlation between IERS and nonsynonymous mutations. **(B)** Forest plot showed statistically significant differences in driver mutated genes between the high IERS and low IERS groups. **(C)** Oncoplot of high-frequency mutated genes between the high- and low-IERS groups. **(D)** Five significant mutation signatures were identified in the high IERS group. **(E)** Four significant mutation signatures were identified in the high IERS group. **(F)** The bar plot demonstrated the CNV events on different chromosome arms of the high- and low-IERS groups. Box plots and scatter plots showed the correlation of IERS and **(G)** amplification counts and **(H)** deletions counts. *P<0.05, **P<0.01, ***P<0.001, ****P<0.0001.

The tumor mutational load (TMB) reflects the number of cancer mutations, some of which can be processed into neoantigens and processed by antigen-presenting cells and presented to T cells to activate the immune response (31, 32). However, neoantigens are not accurately identified when immune escape occurs, affecting the effectiveness of immunotherapy (31, 32). To explore this phenomenon, we then analyzed the TMB between the high and low IERS groups. The results showed that the high IERS group had a higher number of nonsynonymous mutations (**Figure 7A**). In addition, 11 driver mutators including TP53 were significantly upregulated in the high IERS group (**Figure 7B**). Oncoplots show detailed mutational profiles of driver mutators in high and low IERS groups (**Figure 7C**). We then characterized the mutation signatures of different IERS subgroups. In contrast to the low IERS group, the characteristic mutational signatures of the high IERS are SBS1 and SBS5, and COSMIC annotation showed that they are associated with clock-like features that regulate the cell cycle (**Figure 7D**). In contrast, the specific signature of the low IERS group was SBS6, which was associated with the repair of defective DNA mismatch. CNV events are known to drive tumorigenesis and are associated with immune escape (33, 34). We then found that the high IERS group had significantly more CNV events in most chromosome arms (**Figure 7F**). Similarly, we found that the overall number of CNV amplifications and deletions was significantly and positively correlated with IERS and was upregulated in the high IERS group. (**Figures 7G, H**).

### Effect of IERS on first-line chemotherapy

**Figure 8:**
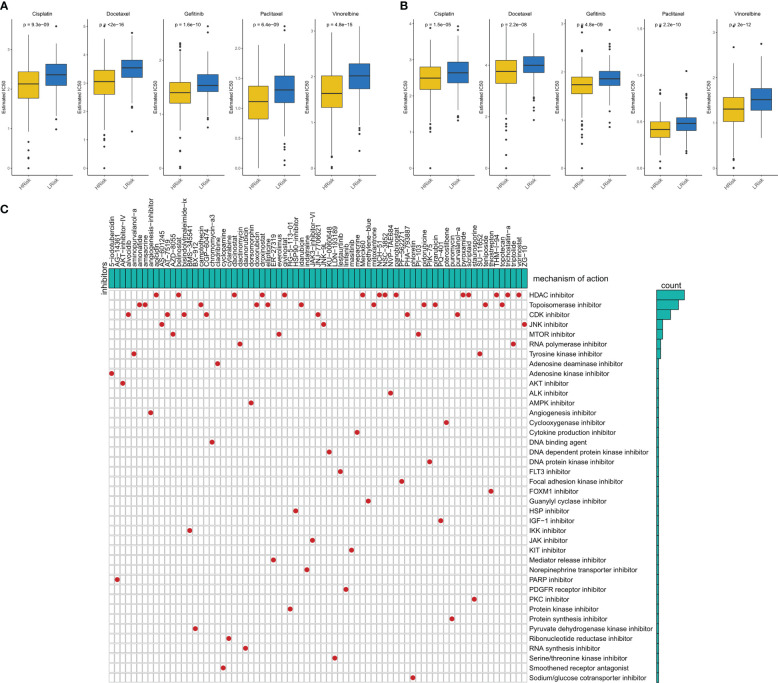
Sensitivity to chemotherapy in different IERS subgroups **(A)** Box plots showed the predicted IC50 values for five first-line drugs of LUAD in high- and low-IERS groups in the TCGA cohort. **(B)** Box plots showed the predicted IC50 values for five first-line drugs of LUAD in high- and low-IERS groups in the meta-GEO cohort. **(C)** Screening potential small molecule compounds for high-risk LUAD patients based on IERS.

We evaluated the sensitivity of five first-line lung cancer chemotherapeutic drugs (Cisplatin, Docetaxel, Gefitinib, Paclitaxel, and Vinorelbine) in different IERS groups. We found that patients with high IERS in the TCGA cohort were more sensitive to all five chemotherapeutic agents (**Figure 8A**), and this result was confirmed in the GEO cohort (**Figure 8B**). Therefore, we hypothesized that patients with high IERS are more suitable for chemotherapy. To explore more possible chemotherapeutic agents, we identified IERS-related small molecule compounds through the Cmap database (**Figure 8C**). A total of 75 small molecules were identified, the most frequent of which is HDAC inhibitor, which may serve as a novel drug to target immune escape in LUAD.

### IERS can predict immunotherapy response

**Figure 9:**
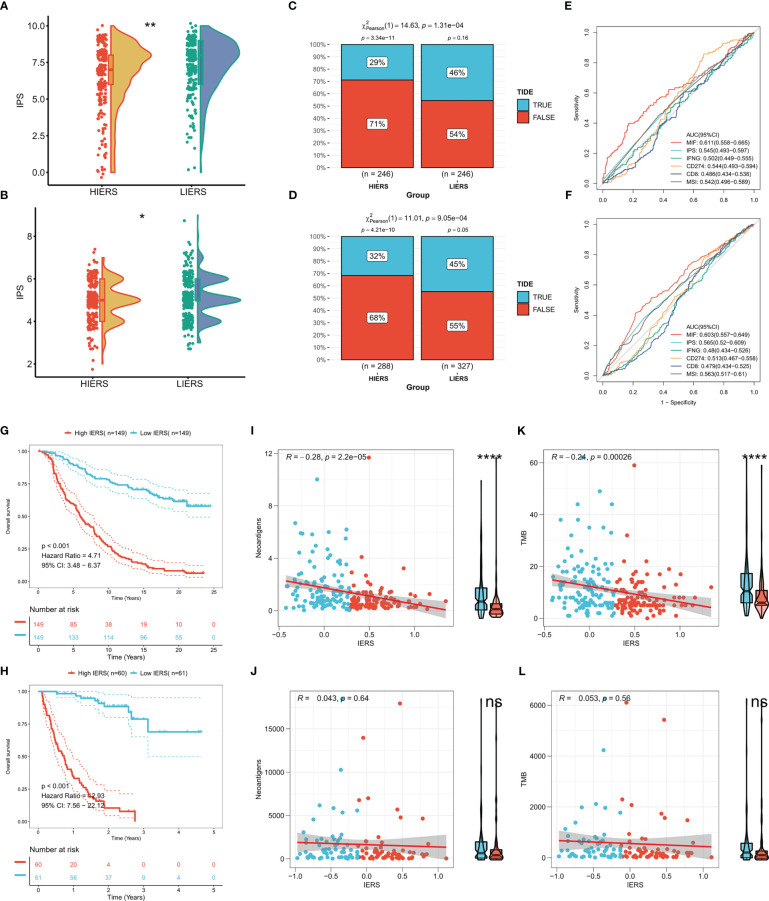
Potential of IERS to predict immunotherapy The boxplot showed the distribution of IPS between different IERS subgroups in the **(A)** TCGA and **(B)** meta-GEO cohorts. TIDE algorithm predicts response to immunotherapy of different IERS subgroups in **(C)** TCGA and **(D)** meta-GEO cohort. ROC curves assess the predictive accuracy of different immune-related metrics for immunotherapy benefits in the **(E)** TCGA and **(F)** meta-GEO cohorts. KM survival curves for patients in the high- and low-IERS groups in **(G)** Imvigor210 and **(H)** Nature-SKCM cohort. Box plots and scatter plots showed the correlation between IERS and neoantigens in **(I)** Imvigor210 and **(J)** Nature-SKCM cohort. Box plots and scatter plots showed the correlation between IERS and TMB in **(K)** Imvigor210 and **(L)** Nature-SKCM cohort. ns P<0.05, *P<0.05, **P<0.01, ****P<0.0001.

We first calculated the IPS, and the results showed that more individuals with high IPS were distributed among patients with low IERS in both TCGA and GEO cohorts (**Figures 9A, B**). We then predicted the response of patients in the TCGA cohort and GEO cohort to immunotherapy by the TIDE algorithm. The results showed that the response in both cohorts was significantly higher in the low IERS group to immunotherapy (**Figures 9C, D**). Moreover, the high IERS group had higher TIDE scores in both cohorts compared to the low IERS group (**Figures S1A, B**), suggesting more T-cell exhaustion events in patients in the high IERS group. The accuracy of IERS and other immunotherapy indicators in predicting response rates to immunotherapy was assessed by ROC curves, and the results showed that IERS was the best predictor of immunotherapy in both cohorts (**Figures 9E, F**). We also constructed and validated our IERS model in two external immunotherapy cohorts. The results showed significantly worse survival in patients with high IERS in the Imvigor210 and Nature-SKCM cohorts (**Figures 9G, H**). Finally, we evaluated the relationship of IERS with neoantigens and TMB recorded in these two cohorts. The results showed a significant negative association of IERS with neoantigens and TMB in the Imvigor210 cohort (**Figures 9I, K**), which may have led to a better survival status of patients with low IERS. However, we did not find a significant correlation between IERS and neoantigens, and TMB in the Nature-SKCM cohort (**Figures 9J, L**).

### IERS in a pan-cancer perspective

**Figure 10:**
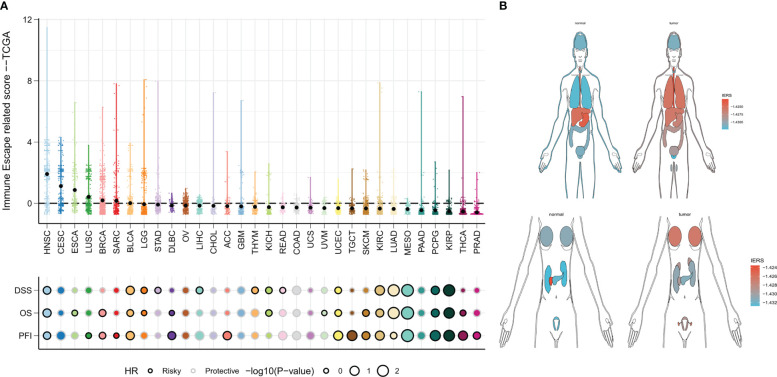
IERS in a Pan-Cancer Perspective **(A)** Distribution and univariate Cox regression analysis of IERS in different solid tumors. **(B)** Distribution of IERS in different organs and tissues.

We finally constructed IERS in the TCGA-pancancer cohort to assess the potential for extrapolation of the final model. The results were satisfactory, with univariate Cox regression showing that IERS can be a reliable predictor of overall survival (OS), Disease Specific Survival (DSS), and Progression Free Interval (PFI) in most solid tumors and can be used as an independent risk factor (**Figure 10A**). In addition, we evaluated the distribution of IERS among different organs and tissues. The results showed that IERS was higher in most of the tumor tissues and may represent the immune escape state at the time of most tumorigenesis (**Figure 10B**). Interestingly, IERS expression was slightly higher in normal tissues of pancreatic and gastric cancers (**Figure 10B**).

### Validation of key IERS model indicator by cellular experiments

**Figure 11:**
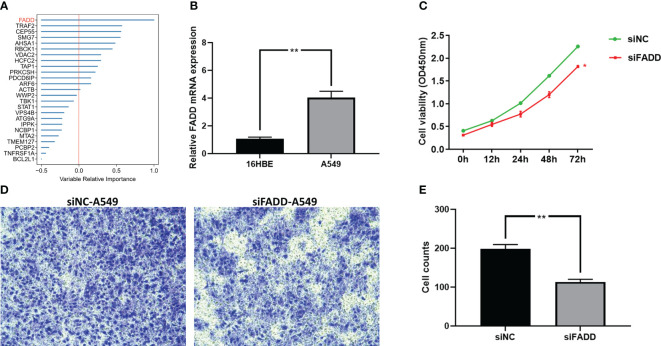
Validation the malignancy of FADD by cellular experiments **(A)** Random forest shows that FADD is the most important variable. **(B)** Differential mRNA expression levels of FADD in 16HBE and A549 cell lines by qPCR. **(C)** Cell proliferation of A549 cells transfected with FADD siRNA or siNC. **(D)** Transwell assay of invasive ability of A549 cells transfected with FADD siRNA or siNC. **(E)** Cell counting of A549 transfected with FADD siRNA or siNC in transwell cells. * P < 0.05, ** P < 0.01.

The final model was screened by the random forest algorithm to incorporate 25 variables, of which FADD was the most important variable in the model (**Figure 11A**). We then explored the effect of FADD on the malignancy of lung adenocarcinoma through *in vitro* experiments. We first found that the mRNA expression level of FADD was increased in LUAD cell lines compared to normal bronchial epithelial cell lines (**Figure 11B**). We then found the reduced proliferative activity of cells after knockdown of FADD in A549 cell line by CCK8 kit (**Figure 11C**). After knockdown of FADD in A549, invasive cells in transwell cells were reduced (**Figure 11D**). By counting the invading cells, we found that the degree of invasion of A549 cells was significantly reduced after knockdown of FADD (**Figure 11E**).

## Discussion

Treatment options for lung cancer are surprisingly evolving, and some LUAD patients can already benefit from novel immunotherapies and improve their prognosis (5). Existing clinical management of lung cancer mainly relies on the traditional AJCC tumor stage, however, traditional stages are increasingly not adapted to the needs of the new world. The introduction of immunotherapy means that patients need better personalized management to aid clinical decision-making (35). Immune escape has been identified as one of the major causes of immunotherapy efficacy, and here we investigated the relationship between immune escape profiles and prognosis and treatment benefit in patients with LUAD.

In this study, we systematically analyzed the transcriptional profile of TCGA-LUAD to characterize and identify prognostic immune escape-related genes in LUAD. The immune escape genes were mainly regulated by CNV but not SNV. TNFRSF1A, TAP1, TRAF2, and PSMB8 were also regulated by methylation. Most of the immune escape genes are risk factors for OS. Based on these 37 prognostic immune escape genes, we developed an integrated pipeline of machine learning to construct an accurate and robust IERS. a total of 101 algorithm combinations passed the pipeline, and we finally confirmed the best combination of RSF+GBM by TCGA and GEO datasets. Our comparison with 10 other published features confirms that our final GBM model has superior performance. A comprehensive meta-analysis of prognosis confirmed IERS as an independent risk factor for OS. Although we observed poor efficacy of IERS in the GEO cohort by ROC curves, this may be due to differences in data standardization and chip platforms. After integrating the TCGA and GEO datasets, we constructed the Nomogram to be used for individual risk stratification in clinical practice. The predictive performance of the Nomogram over the AJCC stage was confirmed by calibration curves, timeROC curves, and DCA curves. In summary, our IERS model has great potential for clinical application.

Precision prediction of lung adenocarcinoma is a hot research area, and previous studies have reported a large number of relevant genetic markers for predicting clinical outcomes in LUAD patients (36–39). Our IERS model has shown a leading edge against 10 published markers. In addition, we evaluated and confirmed the generalizability of the IERS model in a pan-cancer and immunotherapy cohort compared to other published models.

Alteration and activation of the cancer genome led to changes in protein function ultimately causing phenotypic changes (40), therefore we explored the biological functions and TME differences in patients with different IERS status. We observed that higher IERS was associated with a more active cell cycle, DNA metabolism, and cell proliferation. In addition, cell cycle, DNA replication, and homologous recombination pathways were significantly upregulated in the high IERS group. However, we confirmed that asthma, antigen presentation, and hematopoietic cell lineage-related pathways were active in the low IERS group. Therefore, it is reasonable to speculate that the worse OS in high IERS patients is mainly due to the accumulation of immune escape abnormalities resulting in malignant tumor proliferation. To further confirm this hypothesis, we examined the TME components of the high and low IERS subgroups. Convincingly, we found higher tumor purity in high IERS tumor samples and higher immune scores in low IERS tumor samples. Notably, we found that the increased immune cells in the high IERS group were mainly M0 macrophages as well as M2 macrophages. This may have led to a suppressed immune microenvironment in the high IERS group, ultimately causing poorer tumor progression and survival (41). In contrast, monocytes, plasma cells, mast cells, and dendritic cells infiltrated more in the low IERS group. Studies suggest that DC cells and monocytes play an important role in antitumor immunity and may be promising targets for immunotherapy (42, 43). In addition, most antitumor-related immune pathways were active in the low IERS group, such as T-cell co-stimulation, CCR, and check point. We also found that patients with high IERS had higher HRD scores, which may enhance immune escape (33, 34). Finally, we found that low IERS was associated with higher neoantigens. Therefore, it is reasonable to infer that LUAD patients with low IERS have an active TIME of anti-tumor immunity and therefore have a better prognosis and may be more sensitive to immunotherapy.

Our analysis of maf data confirms that IERS can distinguish well between genomic patterns of variation in individual patients. We observed significantly higher TMB in the high IERS group and that most driver mutant genes clustered in high IERS. It is commonly thought that higher TMB may generate more neoantigens, but immune escape in high IERS may hinder neoantigen capture and recognition. Notably, TP53 mutations are increased in patients with high IERS. Studies have shown that TP53 is a popular mutation site in lung cancer and is associated with the malignant progression of LUAD (44, 45). This explains the worse prognosis of patients with high IERS. For the mutational signature, the low IERS group had more SBS6, and previous studies concluded that SBS6 is associated with DNA mismatch repair, and we infer here that SBS may be the set of mutational base features that lead to a better prognosis in LUAD. Interestingly, we found significantly more CNV events in high IERS, and previous studies suggesting homologous recombination as a dominant factor for CNV are consistent with our study (46, 47). In addition, CNV has been suggested to be one of the drivers of immune escape, which may also contribute to immune escape in high IERS (33, 34). In conclusion, we infer that patients with low IERS are more suitable for immunotherapy compared to high IERS.

We confirmed that patients with high IERS are more sensitive to chemotherapy by obtaining drug sensitivity data from the GDSC database. Specifically, we detected that patients with high IERS were more sensitive to Cisplatin, Docetaxel, Gefitinib, Paclitaxel, and Vinorelbine in the TCGA and GEO databases. In addition, we screened for possible drug targets and identified corresponding small molecule compounds for high-risk LUAD patients with high IERS. We identified 75 small molecule compounds, the most common of which is HDAC inhibitor, which may serve as a novel drug to target immune escape in LUAD.

Finally, we predicted that patients with low IERS are more sensitive to immunotherapy from multiple perspectives. First, LUAD patients with low IERS had higher IPS, suggesting that low IERS patients may be more responsive to immunotherapy. In addition, the TIDE algorithm also confirmed that patients with low IERS had higher response rates to immune checkpoint inhibitors (e.g., anti-PD-1, anti-PD-L1, and anti-CTLA-4). Moreover, IERS predicted immunotherapy response rates more accurately than conventional predictors. These findings were also confirmed in an external validation cohort. More convincingly, we demonstrated in the immunotherapy cohort IMvigor210 cohort and Nature-SKCM that IERS is an unfavorable prognostic factor for OS. We found a negative correlation between IERS and neoantigens and TMB, which may explain the better outcome of patients with low IERS for immunotherapy.

The final IERS model can be applied in clinical practice based on a simple PCR assay, however, this study still contains some limitations. First, the final IERS model in this study still contains too many indicators (25), which is not favorable for easy clinical application and cost savings. In addition, the dynamics of the genome is a large field, and we focused on only a portion of the driver immune escape genes, which may overlook some potential associations. Finally, the mechanism by which immune escape affects biological function, as well as phenotype, is unclear, but we combined the results of functional enrichment analysis to make reasonable speculations, which is an inspiration for future mechanistic studies.

In conclusion, based on systematic machine algorithms and large-scale bioinformatics data, we developed and validated a stable and effective immune escape signature for evaluating the prognosis of LUAD patients and response to novel treatment approaches. This IERS model is a promising clinical tool for risk stratification and personalized protocols for patients with LUAD.

## Data availability statement

The original contributions presented in the study are included in the article/**Supplementary material**. Further inquiries can be directed to the corresponding author.

## Author contributions

TW contributed to study design, data analysis, and manuscript writing. LH and JZ contributed to data evaluation and data discussion. LL contributed to the funding and guidance. All authors contributed to the article and approved the submitted version. 

## Supplementary material

Supplementary Figure 1 The TIDE scores in different IERS subgroups **(A)** TIDE scores in TCGA cohort. **(B)** TIDE scores in meta-GEO cohort.
