# Supplementary figures and images for: Systematic integration of machine learning algorithms to develop immune escape-related signatures to improve clinical outcomes in lung adenocarcinoma patients
Source: Front Immunol. 2023 Mar 2;14:1131768. doi: 10.3389/fimmu.2023.1131768 (PMC10018159; doi:10.3389/fimmu.2023.1131768)

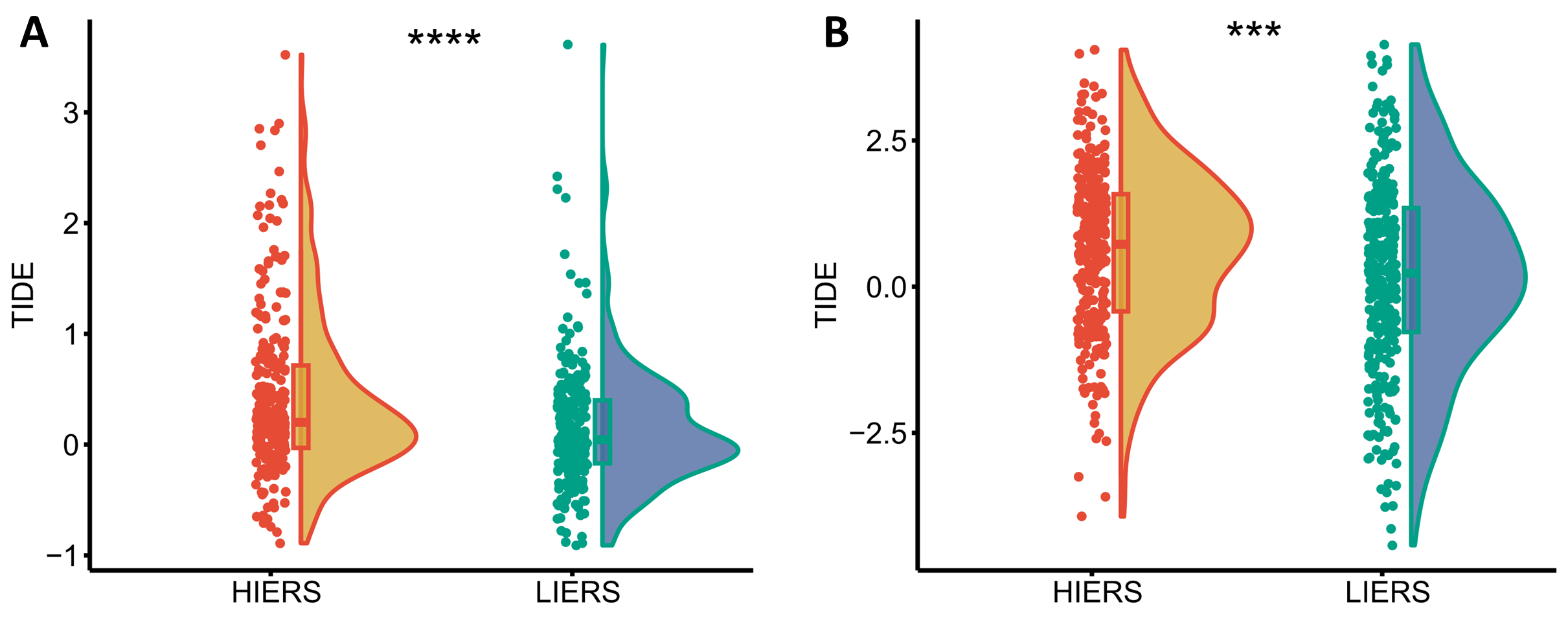

Supplement: Supplementary Figure 1 — The TIDE scores in different IERS subgroups (A) TIDE scores in TCGA cohort. (B) TIDE scores in meta-GEO cohort. [file Image_1.tif]
